# Non-Coding Polymorphisms in Nucleotide Binding Domain 1 in *ABCC1* Gene Associate with Transcript Level and Survival of Patients with Breast Cancer

**DOI:** 10.1371/journal.pone.0101740

**Published:** 2014-07-31

**Authors:** Tereza Kunická, Radka Václavíková, Viktor Hlaváč, David Vrána, Václav Pecha, Karel Rauš, Markéta Trnková, Kateřina Kubáčková, Miloslav Ambruš, Ludmila Vodičková, Pavel Vodička, Pavel Souček

**Affiliations:** 1 Department of Toxicogenomics, National Institute of Public Health, Prague, Czech Republic; 2 3rd Faculty of Medicine, Charles University, Prague, Czech Republic; 3 Department of Oncology, Palacky University Medical School and Teaching Hospital, Olomouc, Czech Republic; 4 Institute for the Care for Mother and Child, Prague, Czech Republic; 5 Biolab Praha, k.s., Prague, Czech Republic; 6 Department of Oncology, Motol University Hospital, Prague, Czech Republic; 7 Department of Radiotherapy and Oncology, Faculty Hospital Kralovske Vinohrady, Prague, Czech Republic; 8 Institute of Experimental Medicine, Czech Academy of Sciences, Prague, Czech Republic; 9 Institute of Biology and Medical Genetics, 1st Faculty of Medicine, Charles University, Prague, Czech Republic; Sudbury Regional Hospital, Canada

## Abstract

**Objectives:**

ATP-Binding Cassette (ABC) transporters may cause treatment failure by transporting of anticancer drugs outside of the tumor cells. Multidrug resistance-associated protein 1 coded by the *ABCC1* gene has recently been suggested as a potential prognostic marker in breast cancer patients. This study aimed to explore tagged haplotype covering nucleotide binding domain 1 of *ABCC1* in relation with corresponding transcript levels in tissues and clinical phenotype of breast cancer patients.

**Methods:**

The distribution of twelve *ABCC1* polymorphisms was assessed by direct sequencing in peripheral blood DNA (n = 540).

**Results:**

Tumors from carriers of the wild type genotype in rs35623 or rs35628 exhibited significantly lower levels of ABCC1 transcript than those from carriers of the minor allele (p = 0.003 and p = 0.004, respectively). The ABCC1 transcript levels significantly increased in the order CT-GT>CC-GT>CC-GG for the predicted rs35626-rs4148351 diplotype. Chemotherapy-treated patients carrying the T allele in rs4148353 had longer disease-free survival than those with the GG genotype (p = 0.043). On the other hand, hormonal therapy-treated patients with the AA genotype in rs35628 had significantly longer disease-free survival than carriers of the G allele (p = 0.012).

**Conclusions:**

Taken together, our study shows that genetic variability in the nucleotide binding domain 1 has a significant impact on the ABCC1 transcript level in the target tissue and may modify survival of breast cancer patients.

## Introduction

Breast cancer (OMIM: 114480) is the most common malignancy affecting female population worldwide. Despite early detection and improved understanding of molecular mechanisms of this disease, it is still the second leading cause of cancer death in women [1].

Multidrug resistance (MDR) represents a major obstacle to successful therapy of tumors. MDR was first described in 1970 [2] as a cross-resistance to structurally and functionally different anticancer drugs. Most MDR is caused by enhanced expression of membrane-bound ATP-Binding Cassette (ABC) transporters [3], [4]. ABC transporters pump drugs outside of the cells into the extracellular space, thus reducing their cytotoxic effect [5]–[7].

Multidrug resistance-associated protein 1 (MRP1/ABCC1, OMIM: 158343) was the first identified member of the ABCC subfamily [8]. *ABCC1* gene is located on the 16^th^ chromosome at position p13.11, is approximately 200 kb long, comprises 31 exons, and encodes 190 kDa membrane protein comprising 1531 amino acids [9]. ABCC1 transports a number of physiological substrates (glutathione, leucotrienes, prostaglandins, etc.) and xenobiotics including anticancer drugs (anthracyclines, taxanes, methotrexate, *Vinca* alkaloids, camptothecins, etc.) [10]. The involvement of ABCC1 in the resistance to chemotherapy has been reported in various types of solid tumors [11].

A recent tissue microarray study has concluded that high ABCC1 protein expression is a negative prognostic marker as it has been found in highly aggressive molecular subtypes of breast carcinoma [12]. Significant overexpression of ABCC1 transcript in both pre-chemotherapy (n = 100) and post-chemotherapy (n = 68) tumors compared with adjacent non-neoplastic tissues from breast carcinoma patients and associations of intratumoral transcript levels with tumor grade and expression of estrogen receptor, proliferative marker Ki67, and p53 protein have been recently reported [13].

**Figure 1:**
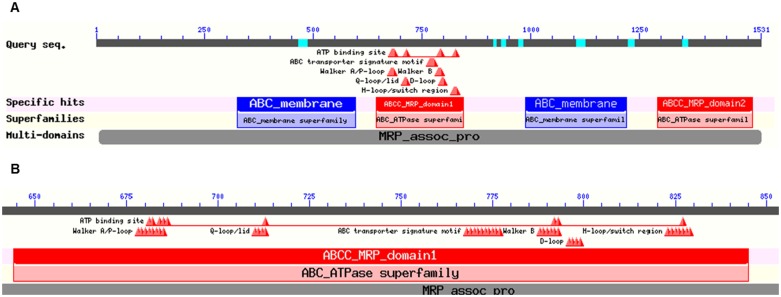
Schematic representation of functional domains of ABCC1. Figure depicts functional domains of ABCC1 protein (A) and important structural motifs within NBD1 (B). Data modified from NCBI’s Conserved Domain Database (CDD) [49].

A high number of single nucleotide polymorphisms (SNPs) in *ABCC1* have been identified in different human populations and their haplotypes were examined [14], [15]. *ABCC1* has high haplotype diversity with significant differences across ethnic groups [16]. Very recently convincing association between rs4148350, rs45511401, and rs246221 SNPs in *ABCC1* and risk of febrile neutropenia in breast cancer patients treated by 5-fluorouracil, epirubicin and cyclophosphamide (FEC regimen) has been shown [17]. Several studies suggested *in vitro* functional effects of SNPs in *ABCC1*. For instance, Gly671Val (dbSNP: rs45511401) SNP located near the nucleotide binding domain 1 (NBD1, **Figure 1**) which is important for the ATPase activity was associated with reduced levels of ABCC1 transcript [14]. Serine at position 433 (rs60782127) significantly increased the resistance to doxorubicin [18] whereas serine at position 43 (rs41395947) enhanced expression and altered ABCC1 protein trafficking to the plasma membrane [19]. Moreover, several *ABCC1* SNPs including Arg723Gln (rs4148356) located between the Walker A and B motifs in NBD1 have been shown to affect the resistance to a number of anticancer drugs [20].

The present study investigated the effect of tagged haplotype of the *ABCC1* gene covering NBD1 with adjacent sequences at ABCC1 transcript level in tumor and non-neoplastic tissues from breast cancer patients. In addition, we also addressed the prognostic and predictive significance of genetic variability of *ABCC1*.

## Materials and Methods

### Material

General chemicals, histopaque (Ficoll), phenol, chloroform, RNase A, proteinase K, *Taq* polymerase, and histidine were purchased from Sigma-Aldrich (Prague, Czech Republic). Deoxynucleotides (dNTPs) for PCR and molecular weight standards for electrophoresis (ΦX174DNA/HaeIII digest) were products of New England Biolabs, Inc. (Ipswich, MA). Ultrapure agarose was supplied by Life Technologies (Prague, Czech Republic).

### Patients

The study included a total of 540 breast cancer patients (C50 according to ICD-10) of Caucasian origin diagnosed in Motol Faculty Hospital, Institute for the Care for Mother and Child, BIOLAB Praha k.s., and Faculty Hospital Kralovske Vinohrady in Prague, Czech Republic between February 2000 and December 2010 (for study flow diagram see **Figure S1 in File S1**). Blood samples were available from all patients. Tumor tissue samples were collected during the primary surgery from subgroups of patients. First subgroup of patients (n = 60) underwent preoperative neoadjuvant chemotherapy regimens based on 5-fluorouracil/anthracyclines/cyclophosphamide (FAC or FEC) and/or taxanes. The second subgroup was treated by adjuvant chemotherapy and/or hormonal therapy after surgery (n = 89). Paired samples of adjacent non-neoplastic tissues as controls were available from 67 patients. In the whole set, patients with metastatic disease treated by first line palliative therapy were also included (for all treatments see **Table S1 in File S1**). Collection and pathological processing of tissue samples and retrieval of data was performed as described before [13], [21]. Expression of receptors for estrogen (ER) and progesterone (PR) was evaluated as positive when at least 10% of cell nuclei showed staining by routine immunohistochemistry. HER2 (ERBB2, OMIM: 164870) status was defined as positive in samples with immunohistochemical score 2+ or 3+ confirmed by SISH analysis. For expression of the p53 (OMIM: 191170) protein, 50% cut off was used (negative <50% vs. positive ≥50%, see Material and Methods S1 and References S1 in File S1). Patients were experimentally divided into groups according to molecular subtypes of their tumors (Luminal A = ER+/HER2− and grade 1 or 2, Luminal B/HER2- = ER+/HER2− and grade 3, Luminal B/HER2+ = ER+/HER2+, HER2+ = ER−/HER2+, and triple negative = ER−/PR−/HER2−) according to [22]. All patients after primary chemotherapy and surgery were followed for local or distant relapse or in the case of palliative setting for disease progression by regular visits every 3 months during the first 3 years, twice a year during the next 2 years and yearly then after. During the visits mammography, chest X ray, skeletal survey, and abdominal ultrasound was performed yearly and clinical examination together with tumor markers (CEA and CA 15-3) was performed during every visit. In the case of clinical uncertainty, additional tests and examinations were performed to rule out possible disease relapse or progression.

### Ethics statement

All patients were asked to read and sign an informed consent and the study was approved by the Ethical Commission of the National Institute of Public Health in Prague.

### DNA extraction

Blood samples were collected during the diagnostic procedures using tubes with K_3_EDTA anticoagulant. Genomic DNA was isolated from human peripheral blood lymphocytes by the standard phenol/chloroform extraction and ethanol precipitation method [23]. DNA samples were stored in aliquots at −20°C prior to analysis.

### ABCC1 genotyping

DNA sequence covering coding exons 15–19 (NBD1), interspersed introns, and sequences surrounding both 5′- and 3′-untranslated regions (Chr16∶16,076,000–16,091,000, NCBI Build 36.3 version) were analyzed by HaploView v4.2 program [24]. Together nine SNPs tagging common haplotypes at r^2^>0.8 and minor allele frequency (MAF)>0.05 in HapMap CEU sample with minimally 75% genotype data were identified. The *ABCC1* region containing eight selected SNPs (rs35623, rs4148351, rs35626, rs11075295, rs3851711, rs3888565, rs35625, and rs4148350) was then divided into four regions. Inside of these regions, we also analyzed additional four SNPs (rs35628, rs11866794, rs4148353, and rs4148356). All analyzed SNPs are characterized in **Table S2 in File S1**. For each region pair of forward and reverse primers with M13 sequence adaptors was designed using the Primer3 software [25]. Oligonucleotide primers were synthesized by Generi Biotech (Hradec Kralove, Czech Republic). Twelve SNPs were then determined by direct sequencing. PCR products were generated using 50 ng of genomic DNA in a 25 µl final volume containing 2.5 µl of 10× reaction buffer consisting of 0.8 (region 1) or 1.6 mM MgCl_2_ (regions 2–4), 0.25 mM dNTPs, 0.2 µM of each primer, and 0.5 µl of *Taq* DNA polymerase, 1 U/µl (all chemicals except for dNTPs from Top-Bio, Vestec, Czech Republic). Primer sequences and optimized conditions for PCR cycling are specified in **Table S3 in File S1**. The PCR products were resolved and analyzed on 2% agarose gel containing ethidium bromide and visualized by ultraviolet light. All samples containing the PCR products were then sequenced by using the BigDye Terminator v3.1 Cycle Sequencing Kit (Life Technologies) with 5 ng of PCR product and 2 pmol of universal M13 sequencing primer in a 10 µl final reaction volume. PCR conditions for sequencing reactions were as recommended by the producer (Life Technologies). Separate sequencing reaction included a control template pGEM-3Zf(+) under the same conditions as above. Sequencing products were purified by EDTA/sodium acetate/ethanol precipitation. DNA sequencing was performed on Applied Biosystems 3130×L Genetic Analyzer and the results were evaluated by Sequencing Analysis Software v5.2 (Life Technologies). About 10% of samples were re-sequenced with 100% conformity of the results.

### ABCC1 gene expression

Total RNA was isolated from frozen tissues, stored, and characterized as described [21]. cDNA was synthesized using 0.5 µg of total RNA and random hexamer primers with the help of RevertAid™ First Strand cDNA Synthesis Kit (MBI Fermentas, Vilnius, Lithuania). Quality of cDNA in terms of DNA contamination was confirmed by PCR amplification of *ubiquitin C* [26]. Quantitative real-time PCR (qPCR) of ABCC1 and reference genes EIF2B1 (OMIM:606686), MRPL19 (OMIM:611832), IPO8 (OMIM:605600), and UBB (OMIM:191339) was performed in RotorGene 6000 (Corbett Research, Sydney, Australia) as described [13]. Reference genes for data normalization were selected using software programs geNorm (version 3.5) and NormFinder (version 19) (see File S1). The qPCR study design adhered to the MIQE Guidelines (Minimum Information for Publication of Quantitative Real-Time PCR Experiments) [27].

### Statistical analyses

The following differences in distribution of genotypes were evaluated: wild type *vs.* minor allele carrier (dominant model) and rare genotype carrier *vs*. wild type allele carrier (recessive model). The additive model was also tested. Haplotypes were evaluated using HaploView software program version 4.2 [24]; phasing of haplotypes prior to a block selection was done using the E-M algorithm and the block selection was based on confidence intervals [28]. Associations between categorized values as genotypes or haplotypes and clinical-pathological data were analyzed using the two-sided Fisher’s Exact test. Clinical and pathological variables included menopausal status (pre- *vs.* post- or perimenopausal), stage (stage I *vs*. stage II–IV), tumor size (pT1 *vs*. pT2-4), lymph node metastasis (pN0 *vs.* pN1-3), histological type (invasive ductal *vs.* other invasive carcinoma) and grade (grade 1 *vs.* grade 2 or 3), expression of ER, PR, and HER2 (negative *vs.* positive), p53 expression (negative *vs.* positive), and molecular subtypes (triple negative *vs*. other and luminal A *vs.* luminal B/HER2−). Differences in transcript levels or age between patients divided by categorized data as genotypes, haplotypes, and clinical-pathological data were evaluated by nonparametric tests (Mann-Whitney, Kruskal-Wallis). Disease-free survival (DFS) was evaluated by the Kaplan-Meier method and the Breslow test was used for evaluation of the compared groups of patients. Multiparametric analysis was then performed by the Cox proportional hazards model. DFS was defined as the time elapsed between surgical treatment and disease progression or death from any cause. Patients lost to follow-up and patients with stage IV disease were excluded from DFS analyses. The results were evaluated by the statistical program SPSS v15.0 (SPSS, Chicago, IL). All p-values are departures from two-sided tests. A p-value of less than 0.05 was considered statistically significant. The correction for false discovery rate (FDR) was applied according to Benjamini and Hochberg [29] and q-values are provided for each comparison. The functional relevance of examined SNPs was analyzed *in silico* by Regulome DB (http://regulome.stanford.edu), PolyPhen-2 (http://genetics.bwh.harvard.edu/pph2), and SIFT (http://sift.jcvi.org) programs. Genetic variants and their observed associations with clinical and functional phenotype were submitted to NCBI (The National Center for Biotechnology Information) ClinVar database (http://www.ncbi.nlm.nih.gov/clinvar).

## Results

### Patients’ characteristics

Clinical characteristics of patients are presented in **Table 1**.

**Table 1:** Clinical-pathological characteristics of patients.

Characteristics	Type	n	%
**Average age at diagnosis**	58±11 years	540	100.0
**Menopausal status**	premenopausal	119	22.2
	postmenopausal	416	77.8
	not assessed	5	–
**Histological tumor type**	invasive ductal	400	76.0
	other invasive type	126	24.0
	not assessed	14	–
**Histological grade (G)**	GI	103	22.1
	GII	238	51.1
	GIII	125	26.8
	Gx	74	–
**Stage (S)**	SI	223	44.7
	SII	211	42.3
	SIII	51	10.2
	SIV	14	2.8
	not assessed	41	–
**pT**	pT1	316	61.5
	pT2	161	31.3
	pT3	17	3.3
	pT4	20	3.9
	pTx	26	–
**pN**	pN0	316	62.0
	pN1	158	31.0
	pN2	25	4.9
	pN3	11	2.2
	pNx	30	–
**cM**	cM0	501	97.1
	cM1	15	2.9
	cMx	24	–
**Expression of estrogen**	positive	393	74.9
**receptor**	negative	132	25.1
	not assessed	15	–
**Expression of**	positive	385	73.8
**progesterone receptor**	negative	137	26.2
	not assessed	18	–
**Expression/amplification**	positive	120	25.2
**of HER2**	negative	357	74.8
	not assessed	63	–
**p53 protein expression**	positive	35	29.9
	negative	82	70.1
	not assessed	423	–

### Distribution of genotypes and haplotypes

Twelve SNPs selected with the help of HaploView v4.2 pairwise tagging algorithm of the region including NBD1 and surrounding sequences of *ABCC1* were genotyped in 540 breast carcinoma patients. The rate of missing genotype data due to DNA of insufficient quality or quantity did not exceed 3.5% in particular SNPs.

**Figure 2:**
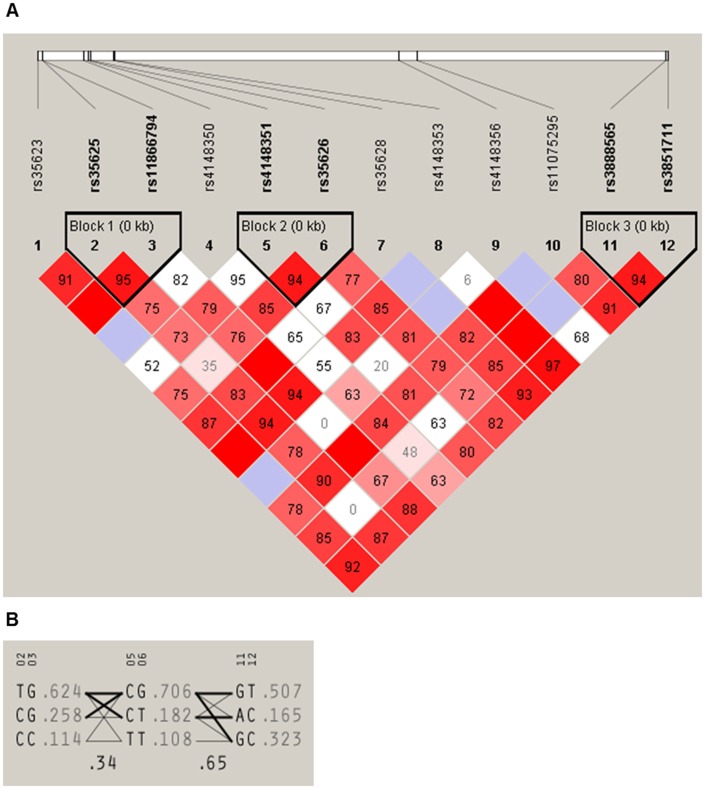
Haplotype analysis of *ABCC1* SNPs. Figure indicates linkage disequilibrium plot (A) and three blocks comprising of SNP diplotypes (B) predicted from experimental data obtained in the present study. The likelihood of linkage of two tested SNPs increases from white to red color (A). Population frequency of diplotypes and connections from one diplotype block to the next one are shown (B). Analysis was performed by HaploView v4.2 program.

The distribution of all analyzed SNPs (rs35623, rs4148351, rs35626, rs11075295, rs3851711, rs3888565, rs35625, rs4148350, rs35628, rs11866794, rs4148353, and rs4148356) is presented in **Table 2**. Genotype distribution of the studied SNPs did not significantly deviate from the Hardy-Weinberg equilibrium (p>0.01). MAFs of these SNPs did not substantially differ from HapMap-CEU population (n = 226) available in dbSNP. Experimental data were reanalyzed by HaploView v4.2 and LD’ values and haplotype blocks were predicted (**Figure 2**). This analysis revealed several SNP-SNP combinations (diplotypes, **Table 3**). To reach reasonable statistical power the most frequent diplotypes with n>40 (highlighted in **Table 3**) were further analyzed.

**Table 2:** Distribution of *ABCC1* SNPs and allele frequencies in breast cancer patients.

SNP ID	Localization	Genotype	distribution		Missing	Type*	MAF#
					genotypes		
		Genotype	n	%	n (%)		
**rs35623**	intron 15	GG	422	79.5	9 (1.7)	NC	T (0.11)
		GT	100	18.8			
		TT	9	1.7			
**rs35625**	intron 15	TT	217	40.9	9 (1.7)	NC	C (0.37)
		TC	234	44.1			
		CC	80	15.1			
**rs11866794**	intron 15	GG	415	78.2	9 (1.7)	NC	C (0.12)
		GC	108	20.3			
		CC	8	1.5			
**rs4148350**	intron 15	GG	466	89.3	18 (3.3)	NC	T (0.06)
		GT	52	10.0			
		TT	4	0.8			
**rs4148351**	intron 15	CC	416	79.5	17 (3.1)	NC	T (0.11)
		CT	97	18.6			
		TT	10	1.9			
**rs35626**	intron 16	GG	261	50.1	19 (3.5)	NC	T (0.29)
		GT	220	42.2			
		TT	40	7.7			
**rs35628**	intron 16	AA	441	84.0	15 (2.8)	NC	G (0.09)
		AG	77	14.7			
		GG	7	1.3			
**rs4148353**	intron 16	GG	426	81.3	16 (3.0)	NC	T (0.10)
		GT	91	17.4			
		TT	7	1.3			
**rs4148356**	exon 17	GG	516	96.3	4 (0.7)	R723Q	A (0.02)
		GA	19	3.5			
		AA	1	0.2			
**rs11075295**	intron 17	AA	375	69.8	3 (0.6)	NC	G (0.17)
		AG	139	25.9			
		GG	23	4.3			
**rs3888565**	intron 18	GG	380	70.6	2 (0.4)	NC	A (0.17)
		GA	134	24.9			
		AA	24	4.5			
**rs3851711**	intron 18	TT	151	28.1	2 (0.4)	NC	G (0.49)
		TC	248	46.1			
		CC	139	25.8			

Footnotes:

*NC = non-coding.

# MAF = minor allele frequency.

**Table 3:** Distribution of *ABCC1* diplotypes predicted by HaploView v4.2.

Diplotype 1		rs11866794		
		GG	GC	CC
rs35625	TT	**215**	1	1
	TC	**161**	**73**	0
	CC	39	34	7
**Diplotype 2**			rs35626	
		GG	GT	TT
rs4148351	CC	**258**	**140**	17
	CT	2	**80**	14
	TT	1	0	9
**Diplotype 3**			rs3851711	
		TT	TC	CC
rs3888565	GG	**151**	**163**	**66**
	GA	0	**81**	**53**
	AA	0	4	20

Numbers of patients with combinations of diplotypes presented.

The most frequent diplotypes used for statistical analyses in bold.

### Associations of ABCC1 SNPs and diplotypes with transcript levels

**Figure 3:**
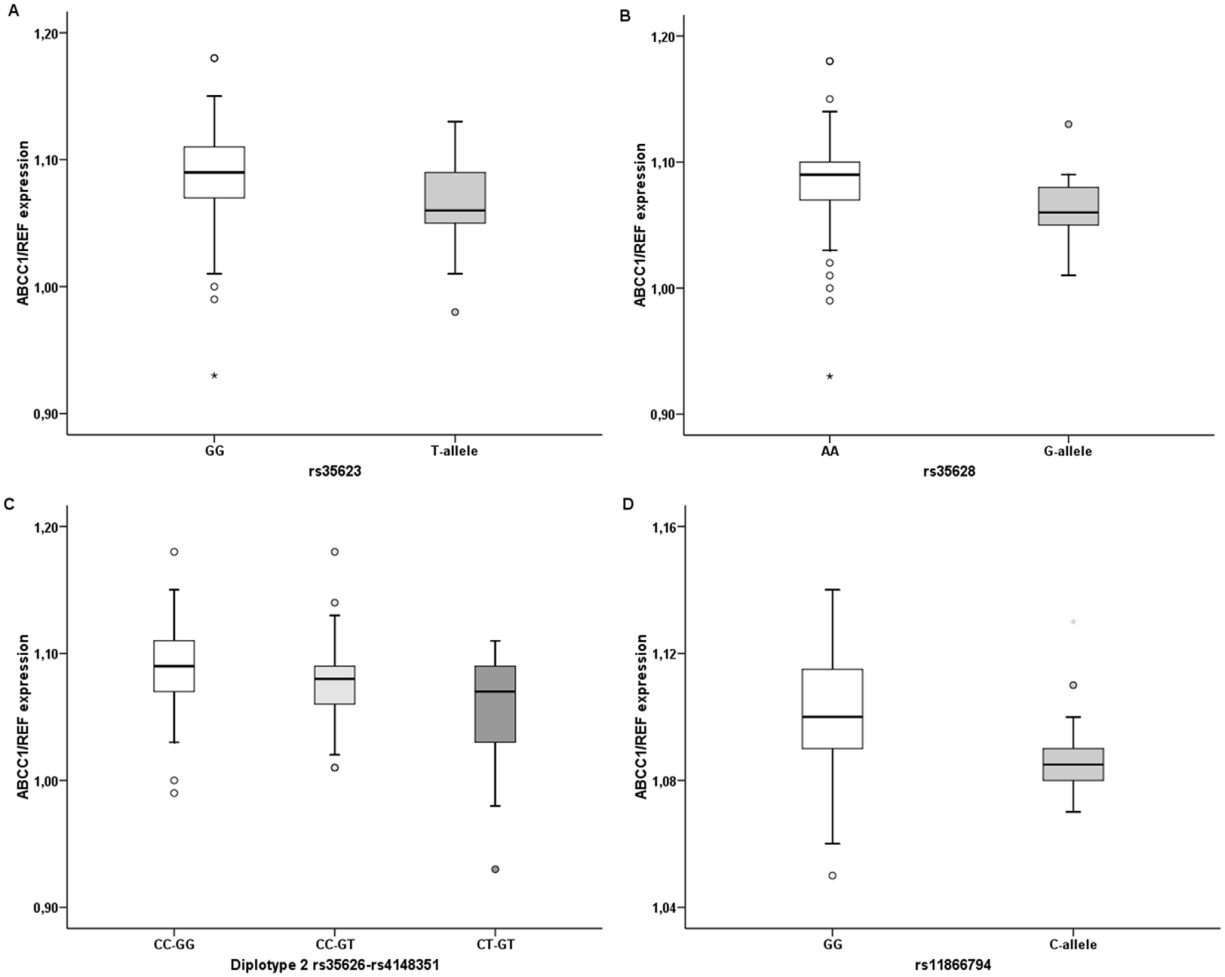
Significant associations between transcript levels and polymorphisms in *ABCC1*. All SNPs and frequent diplotypes were analyzed but to retain concise style only significant associations are reported.

The ABCC1 transcript level was previously assessed in tumors and non-neoplastic control tissues from breast cancer patients [13]. A subset of these patients with complete genotype data was included into this study (n = 149) and associations between genotypes, predicted diplotypes, and transcript levels were analyzed by Mann-Whitney or Kruskal-Wallis tests. Associations of all SNPs and frequent diplotypes with expression levels were analyzed but to retain concise style only significant results are reported (**Table 4** and **Figure 3**). Tumors from carriers of the wild type genotype in rs35623 or rs35628 expressed significantly lower ABCC1 transcript levels than those with the minor allele (p = 0.003 and p = 0.004, respectively; q = 0.008, both significant; **Table 4** and **Figures 3A, B**). A significant upward trend in the ABCC1 transcript level in the order CT-GT>CC-GT>CC-GG (p = 0.023; q = 0.017, non-significant; **Table 4** and **Figure 3C**) for the rs35626-rs4148351 diplotype was observed. Non-neoplastic control tissues from carriers of the wild type genotype in rs11866794 expressed lower ABCC1 transcript levels than those with the minor allele (p = 0.017; q = 0.004, non-significant; **Table 4** and **Figure 3D**). The rs4148356 SNP was predicted to be benign with a score of 0.014 by PolyPhen-2 and tolerated with a score 0.30 by SIFT programs. From synonymous SNPs, rs35626 was classified as likely to affect binding and linked to expression of a gene target (score 1f), rs35625, and rs11866794 as likely to affect binding (2c) by the Regulome DB program. The rest of SNPs was classified as having minimal binding evidence (4–6; **Table S4 in File S1**).

**Table 4:** Significant associations of *ABCC1* polymorphisms with expression levels.

Genotype	n	Normalized ABCC1 expression in tumors (Mean Rank)*
**rs35623**		
GG	116	78.1
GT or TT	29	52.5
Missing	4	–
p-value		0.003#
**rs35628**		
AA	117	73.6
AG or GG	21	46.8
Missing	11	–
p-value		0.004#
**Diplotype 2 rs35626-rs4148351**		
CC-GG	66	69.3
CC-GT	38	60.0
CT-GT	20	44.7
Missing	25	–
p-value		0.023
**Genotype**	**n**	**Normalized ABCC1 expression in controls (Mean Rank)** *
**rs11866794**		
GG	48	36.9
GC or CC	18	24.4
Missing	1	–
p-value		0.017

All SNPs and frequent diplotypes were analyzed but to retain concise style only significant associations are reported.

Footnotes:

*Analyzed by Mann-Whitney test. The higher is the rank the lower is the normalized expression ABCC1/reference genes.

# Result passed FDR analysis for multiple testing [29].

### Associations between clinical characteristics, therapy outcome, and ABCC1 SNPs and diplotypes

Associations of all SNPs and frequent diplotypes with clinical data were analyzed but to retain concise style only significant results are reported (**Table 5**). The *ABCC1* SNP rs3888565 was significantly associated with expression of estrogen receptor (**Table 5**). Carriers of the AA genotype had more frequently tumors without ER expression than carriers of the G allele (p = 0.003; q = 0.004, significant). Moreover, G allele in this SNP was associated with triple-negative disease exhibiting the worst prognosis of all molecular subtypes of breast carcinoma (p = 0.008; q = 0.004, non-significant). Regarding rs4148350, patients with stages II–IV (advanced disease) or lymph nodes affected by metastasis had a greater incidence of the T allele than those with early stage I or metastasis-free lymph nodes (p = 0.005 and p = 0.028, respectively; q = 0.004 and q = 0.008, both non-significant). Similarly, patients with HER2-positive tumors carried more frequently the T allele in rs4148350 than those without HER2 expression (p = 0.014; q = 0.004, non-significant). The T allele in rs4148353 also predisposed patients to tumors with ER expression in comparison with wild type carriers (p = 0.049; q = 0.004, non-significant). On the other hand, tumors of the T allele carriers in respect to rs4148353 were usually HER2-negative (p = 0.001; q = 0.004, significant; **Table 5**). Advanced stages II–IV, similarly as tumors with grades 2 or 3 occurred more frequently in carriers of the C allele in rs35625 than in those with the wild type TT (p = 0.040, p = 0.029, respectively; q = 0.004 and q = 0.008, both non-significant). Carriers of the C allele in rs3851711 had more frequently tumors of histological type other than ductal and exhibited more frequently triple-negative molecular subtype histology than those with the TT genotype (p = 0.040 and p = 0.039, respectively; q = 0.004 and q = 0.008, both non-significant).

**Table 5:** Significant associations of *ABCC1* polymorphisms with clinical data.

Characteristics	rs3888565		p-value*
	GG/GA	AA	
ER negative	119	13	
ER positive	380	10	0.003#
Triple negative	49	7	
Other subtype	465	17	0.008
**Characteristics**	**rs4148350**		**p-value** *
	**GG**	**GT/TT**	
Stage II–IV	229	40	
Stage I	199	14	0.005
pN1-3	162	29	
pN0	276	26	0.028
HER2 negative	315	29	
HER2 positive	96	20	0.014
**Characteristics**	**rs4148353**		**p-value** *
	**GG**	**GT/TT**	
ER negative	111	16	
ER positive	304	78	0.049
HER2 negative	270	76	
HER2 positive	106	10	0.001#
**Characteristics**	**rs35625**		**p-value** *
	**TT**	**TC/CC**	
Grade 2 or 3	133	222	
Grade 1	51	51	0.029
Stage II–IV	101	46	
Stage I	102	26	0.040
**Characteristics**	**rs3851711**		**p-value** *
	**TT**	**TC/CC**	
Ductal type	121	277	
Other type	26	100	0.040
Triple-negative	11	21	
Other subtype	140	118	0.039

All SNPs and frequent diplotypes were analyzed but to retain concise style only significant associations are reported.

Footnotes:

*Analyzed by two-sided Fisher’s Exact test.

# Result passed FDR analysis for multiple testing [29].

**Figure 4:**
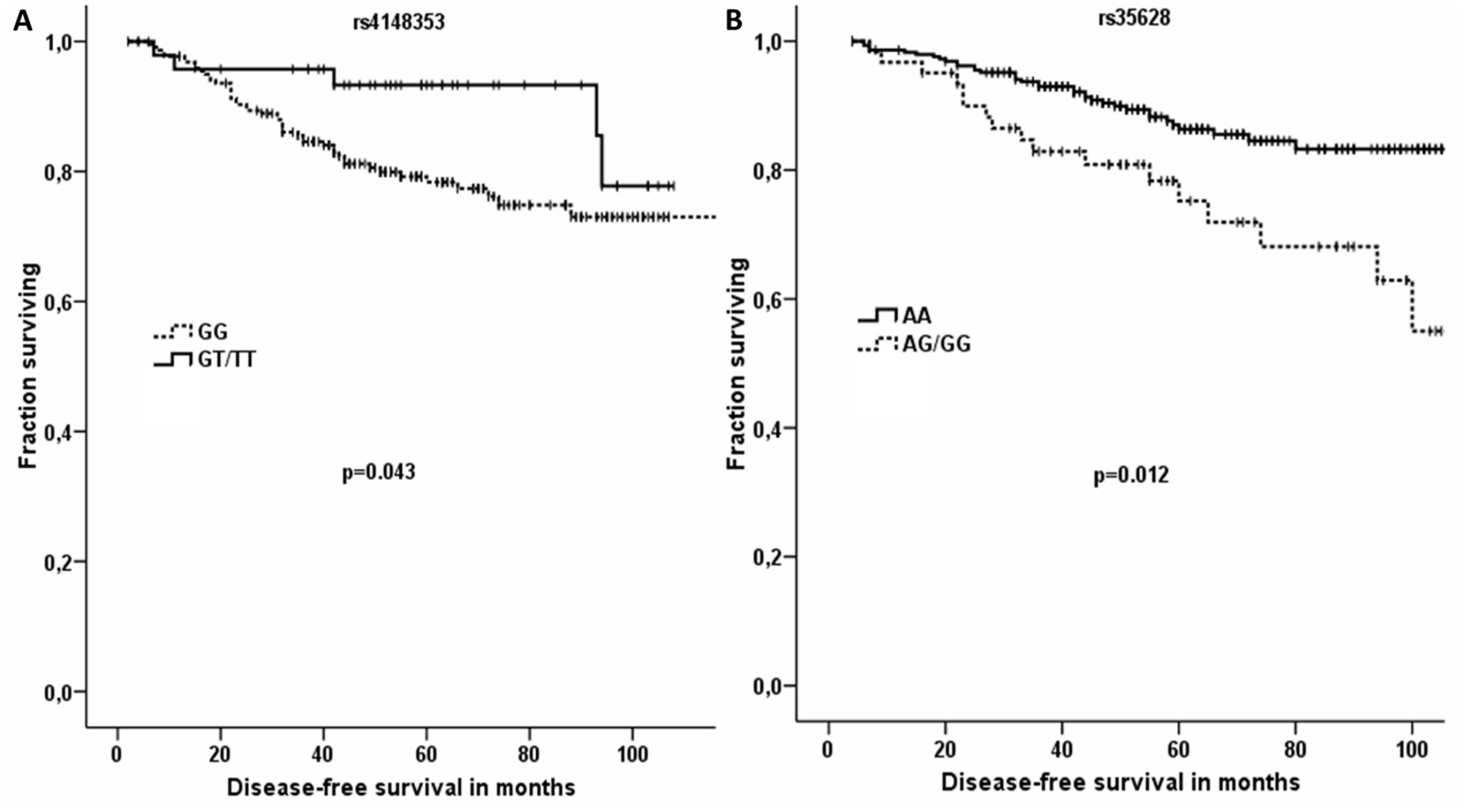
Significant associations between DFS of patients with breast carcinoma and SNPs in *ABCC1*. Kaplan-Meier survival curves for patients treated by chemotherapy (A) and hormonal therapy (B) were analyzed by Breslow test. In part A, dashed line represents DFS of patients with the GG genotype in rs4148353, while solid line indicates that of patients with the T allele. In part B, dashed line represents DFS of patients with the G allele in rs35628 and solid line DFS of those with the AA genotype. All SNPs have been analyzed but to retain concise style only significant associations are reported.

No association between age at diagnosis, menopausal status, tumor size, expression of progesterone receptor, and p53 and the SNPs followed was found (results not shown). Large tumor size (pT2-4), presence of lymph node metastasis (pN1-3), lack of expression of hormonal receptors (ER and PR), and triple-negative molecular subtype were significant predictors of poor prognosis, i.e. short DFS in the set of chemotherapy-treated patients (p<0.001, p = 0.001, p = 0.011, p = 0.001, and p = 0.003, respectively). Large tumor size (pT2-4), presence of lymph node metastasis (pN1-3), and lack of expression of PR were significant predictors of poor prognosis, i.e. short DFS in the set of hormonal therapy-treated patients (p = 0.001, p<0.001, and p = 0.031, respectively). Chemotherapy-treated patients carrying T allele in the rs4148353 SNP had longer DFS than those with wild type GG genotype in univariate analysis (n = 271, p = 0.043; **Figure 4A**). On the other hand, hormonal therapy-treated patients with the wild type AA genotype in the rs35628 had longer DFS than patients carrying the G allele (n = 353, p = 0.012; **Figure 4B**). Multivariate analysis using the Cox regression hazard model with pT, pN, ER, and PR expression, triple-negative molecular subtype, and individual SNPs has not confirmed association with DFS for rs4148353 (n = 252, p = 0.116). However, for rs35628 the association observed in univariate model remained significant in multivariate model with pT, pN, and PR expression (n = 323, p = 0.008). Survival analysis was not corrected for multiple testing.

## Discussion

Multidrug resistance frequently causes cancer treatment failure. Numerous *in vitro* and *in vivo* data revealed that multidrug resistance is often due to enhanced expression ABC transporters [30]. Thus, in depth analysis of ABC transporters appears inevitable for individualization of treatment.

The multidrug resistance-associated protein 1 encoded by the *ABCC1* gene is one of the most studied ABC transporters. Very recently, we demonstrated significant overexpression of ABCC1 transcript in tumors compared to adjacent non-neoplastic tissues from breast cancer patients and suggested its intratumoral levels as potential modifiers of breast carcinoma progression [13]. Another contemporary study has suggested that a high ABCC1 protein expression is a negative prognostic marker, as it has been found in highly aggressive molecular subtypes of breast carcinoma [12]. Despite already accumulated knowledge on ABCC1 there are significant gaps in understanding its role in cancer therapy and prognosis which preclude clinical applications.

NBD1 of ABCC1 contains several functional motifs, ATP-binding site, Walker A/P-loop, Q-loop/lid, ABC transporter signature, Walker B, D-loop, and H-loop/switch (**Figure 1**). Unlike most ABCC proteins, NBD1 of ABCC1 binds ATP with high affinity but has low hydrolytic activity, while the reverse is true of NBD2 [31]. Besides this functional asymmetry, it seems obvious that NBD1 and NBD2 cooperate together and with surrounding transmembrane cytoplasmic loops may influence substrate selectivity and the proper assembly and trafficking of ABCC1 to the plasma membrane [32], [33]. Moreover, very recently, virtual screening of X-ray crystal structure of ABCC1 NBD1 [34] revealed that about 5% of the National Cancer Institute compounds possessed lower docking scores than ATP in ABCC1 NBD1 and it has been suggested that the compounds identified may be potential inhibitors of ABCC1 and require further pharmacological analysis [35]. Apparently, the role of ABCC1 as predictive biomarker and potential drug target in human cancers raises further interest.

The present study addressed yet unexplored associations between genetic variability in NBD1 and adjacent sequences of *ABCC1* and clinical course of breast cancer. Further, it evaluated relations between genotype and phenotype represented by transcript levels in tissues of breast cancer patients.

Analogously with the ABCB1/P-glycoprotein [36], in the present study, we have observed associations between genetic variability in *ABCC1* and its transcript levels in tissues from breast cancer patients. Carriers of the wild type genotype in rs35623 or rs35628 SNPs had significantly lower ABCC1 levels in their tumors than the rest of patients, suggesting their potential as predictors of treatment outcomes. The association of the rare CT-GT diplotype rs35626-rs4148351 with ABCC1 transcript levels observed in the uncorrected analysis has not passed the FDR test and should be replicated on a larger sample size.

The analysis of associations between *ABCC1* SNPs and transcript levels could be confounded by the fact that some patients received pre-operative chemotherapy. Some ABC transporters may be induced by chemotherapy [37]. However, in our study the difference in ABCC1 transcript levels between post- and pre-treatment patients was non-significant (p>0.05).

Most interestingly, the rs35628 SNP significantly influenced DFS of patients treated by hormonal therapy in both univariate and multivariate analysis. Taken together, patients with the wild type genotype AA in rs35628 SNP had lower ABCC1 levels in tumors and better survival rates after hormonal therapy than those with the G allele. Cell lines with overexpression of ABCC1 are resistant to anticancer drugs [30] and high expression of ABCC1 protein was associated with shorter DFS [38]. The role of ABCC1 in the efflux of anticancer drugs has been recently proposed [39], [40]. On the other hand, the intratumoral ABCC1 transcript level did not modify DFS of unselected patients (n = 88) or patients stratified according to the therapy type, referring to a more complex phenomenon [13]. We also have not found significant association between transcript and protein levels of ABCC1 (n = 30) in the previous study on independent set of patients [13].

Tamoxifen metabolites endoxifen and 4-hydroxy-tamoxifen are substrates of the ABCB1 transporter *in vitro* [41], but the association of genetic variation in *ABCB1* and tamoxifen effectiveness is unknown. The role of ABCC1/MRP1 in the transport of tamoxifen, its metabolites or aromatase inhibitors also remains vastly unexplored [42] and thus it is currently impossible to draw any conclusive remarks from our observations in hormonally-treated breast cancer patients.

We have also found out that chemotherapy-treated carriers of the T allele in rs4148353 SNP had significantly better DFS than those with the wild type GG genotype. However, this association has not been confirmed by multivariate analysis. The association of rs4148353 with DFS could be modulated by the significant associations of this SNP with ER and mainly HER2 which were shown to be the best predictors of chemotherapy response in breast carcinoma [43]. Patients carrying the T allele had higher frequency of ER-positive or HER2-negative tumors when compared with wild type carriers in the present study and the lack of expression of hormonal receptors or the triple-negative molecular subtype of breast cancer were indeed significant predictors of poor DFS.

Despite the fact that the *ABCC1* rs4148356 SNP located between the Walker A and B motifs in NBD1 has been shown to affect resistance to a number of anticancer drugs [20], we did not find association of this SNP with DFS in breast cancer patients. We confirmed the previously observed lack of effect of rs4148356 (R723Q, 2168G>A) on ABCC1 expression [44]. Also *in silico* analyses performed by PolyPhen-2 [45] and SIFT [46] programs support our observations. Ten other non-synonymous SNPs leading to amino acid substitutions (Cys43Ser (G128C, rs41395947), Thr73Ile (C218T, rs41494447), Ser92Phe (C257T, rs8187844), Thr117Met (C350T, no rs number available), Arg230Gln (G689A, rs8187848), Arg633Gln (G1898A, rs112282109), Ala989Thr (G2965A, rs35529209), Cys1047Ser (G3140C, rs13337489), Arg1058Gln (G3173A, rs41410450), and Ser1512Leu (C4535T, rs369410659)) followed earlier had no effect on ABCC1 expression either, indicating that single amino acid substitutions may not necessarily influence the activity of the final protein [44]. No significant effect of the synonymous SNPs G816A (rs2230669), T825C (rs246221), T1684C (rs35605), and G4002A (rs2230671) on ABCC1 transcript level in peripheral CD4+ cells has been observed as well [15]. From the synonymous SNPs followed by the present study, *in silico* analyses by help of Regulome DB [47] suggested that rs35625, rs35626, and rs11866794 likely affect regulation of target gene transcription.

The lack of validation study in independent sample set may be seen as limitation of the present study. By searching Catalog of Published Genome-Wide Association Studies at NHGRI (www.genome.gov) and GWAS Central (www.gwascentral.org) we have found no supportive data for associations between *ABCC1* SNPs with breast carcinoma survival or therapy response that could support our results. Microarray study that explored associations of transcript levels with SNP markers from the International HapMap Project in lymphoblastoid cells of 57 unrelated CEPH individuals has not observed such association(s) for ABCC1 [48].

Significant associations of non-coding SNPs with expression and clinical phenotype observed by the present study were not confirmed by *in silico* analyses or additional experimental data. This fact limits the interpretation of the results before complex functional study is completed.

In conclusion, according to our present data, SNPs rs35623 and rs35628 in non-coding regions around NBD1 may modulate ABCC1 transcript levels in breast tumors, thus contributing to a complex pattern of chemotherapy resistance by so far unknown mechanism. Associations of rs35628 and rs4148353 with DFS of breast cancer patients warrant further studies aimed at validation or disqualification of these putative prognostic markers.

## Supporting Information

File S1 Contains the following files: **Material and Methods S1. References S1. Table S1:** Chemotherapy and hormonal therapy regimens. **Table S2:** Positions of the analyzed SNPs in *ABCC1.* **Table S3:** Sequencing primers and PCR conditions for assessment of polymorphisms in NBD1 of *ABCC1.* **Table S4:** *In silico* analysis of functional significance of all studied polymorphisms in NBD1 of *ABCC1.* **Figure S1:** Flow diagram of the study.
(DOC)
