# Supplementary material for: Non-Coding Polymorphisms in Nucleotide Binding Domain 1 in ABCC1 Gene Associate with Transcript Level and Survival of Patients with Breast Cancer
Source: PLoS One. 2014 Jul 31;9(7):e101740. doi: 10.1371/journal.pone.0101740 (PMC4117604; doi:10.1371/journal.pone.0101740)
Supplement: File S1 — Contains the following files: Material and Methods S1. References S1. Table S1: Chemotherapy and hormonal therapy regimens. Table S2: Positions of the analyzed SNPs in ABCC1. Table S3: Sequencing primers and PCR conditions for assessment of polymorphisms in NBD1 of ABCC1. Table S4: In silico analysis of functional significance of all studied polymorphisms in NBD1 of ABCC1. Figure S1: Flow diagram of the study. (DOC) [file pone.0101740.s001.doc]

***Supporting Information***

**Non-coding polymorphisms in nucleotide binding domain 1 in *ABCC1* gene associate with transcript level and survival of patients with breast cancer**

Tereza Kunická^1,2^, Radka Václavíková^1^, Viktor Hlaváč^1,2^, David Vrána^1,3^, Václav Pecha^4^, Karel Rauš^4^, Markéta Trnková^5^, Kateřina Kubáčková^6^, Miloslav Ambruš^7^, Ludmila Vodičková^1,8^, Pavel Vodička^8,9^, Pavel Souček^1*^

*^1^Department of Toxicogenomics, National Institute of Public Health, Prague, Czech Republic*

*^2^3^rd^ Faculty of Medicine, Charles University, Prague, Czech Republic*

*^3^Department of Oncology, Palacky University Medical School and Teaching Hospital, Olomouc, Czech Republic*

*^4^Institute for the Care for Mother and Child, Prague, Czech Republic*

*^5^Biolab Praha, k.s., Prague, Czech Republic*

*^6^Department of Oncology, Motol University Hospital, Prague, Czech Republic*

*^7^Department of Radiotherapy and Oncology, Faculty Hospital Kralovske Vinohrady, Prague, Czech Republic*

*^8^ Institute of Experimental Medicine, Czech Academy of Sciences, Prague, Czech Republic*

*^9^ Institute of Biology and Medical Genetics, 1^st^ Faculty of Medicine, Charles University, Prague, Czech Republic*

# *Corresponding author: Pavel Soucek, PhD, Toxicogenomics Unit, National Institute of Public Health, Srobarova 48, 100 42 Prague 10, Czech Republic, Phone: +420-2 6708 2709, Fax: +420-2 6731 1236, E-mail: psoucek@szu.cz, web: [**www.szu.cz**](http://www.szu.cz/)

# Supplementary Material and Methods

*Patient samples*

After histopathological processing, tissue specimens were snap-frozen in liquid nitrogen. Since this point the pathological processing of the samples differed. In Motol, five-micrometer cryostat sections were prepared for isolation of total RNA. The presence of tumor cells in the sample was histologically verified in the first and in the last section of a row. The sections cut in the layers between the aforementioned histological controls were used for total RNA isolations. Specimens collected in Institute for the Care for Mother and Child were transferred to Biolab for pathological processing. Two specimens, the macroscopically apparent tumorous tissue and the non-tumorous mammary tissue in minimum distance of 20 mm from the tumor, both of 4-5 mm in diameter, were excised from the native tissue sample. One cryostat section from each block was stained by hematoxylin and eosin to confirm the content of tumor cells in both types of specimens. The frozen blocks of tissue were transported in dry ice to the main investigator laboratory for RNA isolation. In both pathology laboratories, the standard processing of surgical sample and diagnostic histological evaluation was followed according to WHO classification [1]. The following data on patients were retrieved from medical records: age, menopausal status, date of diagnosis of breast cancer, personal and family anamnesis (number of relatives affected by breast cancer, ovarian cancer or other malignant diseases), tumor size, lymph node metastasis, clinical stage, histological type and grade of tumor, expression of estrogen, progesterone receptors, and HER-2, expression of p53 protein, therapy, response (in neoadjuvant set only) and disease-free survival.

*Immunohistochemical detection of p53 protein expression*

Fresh tissue samples of the mammary tumors were fixed in standard neutral buffered 4% formaldehyde for up to 26 hours and embedded into paraffin with classical histological techniques. For immunohistochemical investigation 3 µm thick histological sections were utilized. Primary antibody against the p53 (clone DO-7; monoclonal mouse antibody detecting both mutant and wild type p53 protein) was purchased from Dako (Dako, Glostrup, Denmark). Antibodies were diluted with Dako Antibody diluent (1:50). For p53 detection, the sections were further processed with heat-induced epitope retrieval in 10 mmol/l citrate buffer pH 6.0 in water bath (40 min heating at 95-99° C and then 20 min cooling at room temperature). Tissues were incubated with primary antibodies overnight at 4° C. Detection was performed with peroxidase/diaminobenzidine system. Evaluation of binding of both primary antibodies was performed with Dako REAL Detection System (LSAB+, biotinylated secondary goat anti-mouse antibodies/streptavidin conjugated to horseradish peroxidase). As a chromogen, 0.04 % DAB (3,3´-diaminobenzidine tetrahydrochloride dihydrate; Fluka, Buchs, Switzerland) in 50 mmol/l TRIS (Tris-hydroxymethyl amino methane)/0.015 % H_2_O_2_ was used. Several p53 positive cells were present in each sample analyzed. p53 status was evaluated as positive, if more than 50% of tumor cells were immunohistochemically stained according to the previously published evaluation procedure [2, 3].

*Selection of reference genes*

For selection of reference genes, 96-well TaqMan Array Plates (Life Technologies) were used and evaluated as previously published [4, 5]. EIF2B1, MRPL19, IPO8, and UBB were selected as the most stable reference genes for data normalization.

**Supplementary References**

**1. World Health Organization Classification of Tumours. Pathology & Genetics of Tumours of the Breast and Female Genital Organs,** ed. Fattaneh A. Tavassoli & Peter Devilee, IARC Press, Lyon 2003.

2. Kai K, Nishimura R, Arima N, Miyayama H, Iwase N. (2006) p53 expression status is a significant molecular marker in predicting the time to endocrine therapy failure in recurrent breast cancer: a cohort study*. Int J Clin Oncol* **11**:426–433.

3. von Minckwitz G, Sinn HP, Raab G, Loibl S, Blohmer JU, et al. (2008) German Breast Group. Clinical response after two cycles compared to HER2, Ki-67, p53, and bcl-2 in independently predicting a pathological complete response after preoperative chemotherapy in patients with operable carcinoma of the breast. *Breast Cancer Res* **10**:R30.

4. Hlavata I, Mohelnikova-Duchonova B, Vaclavikova R, et al. (2012) The role of ABC transporters in progression and clinical outcome of colorectal cancer. *Mutagenesis* **27**:187-196.

5. Mohelnikova-Duchonova B, Oliverius M, Honsova E, Soucek P. (2012) Evaluation of reference genes and normalization strategy for quantitative real-time PCR in human pancreatic carcinoma. *Dis Markers* **32**:203-130.

**Supporting Information Table S1:** Chemotherapy and hormonal therapy regimens

| **Characteristics** | **Type** | **n** | **%** |
| --- | --- | --- | --- |
| **Neoadjuvant regimen (n=122)** | Anthracycline in combination  Taxane alone  Hormonal therapy  CMF only**^*^** | 112  3  5  2 | 91.8  2.5  4.1  1.6 |
| **Adjuvant or palliative regimen (n=251)** | Anthracycline in combination  Taxane alone  CMF only**^*^** | 177  54  20 | 70.5  21.5  8.0 |
| **Hormonal regimen (n=395)** | Tamoxifen only  Aromatase inhibitors only  Tamoxifen & aromatase inhibitors | 184  105  106 | 46.6  26.6  26.8 |

Footnotes:

^*^CMF=cyclophosphamide/methotrexate/5-fluorouracil combination

**Supporting Information Table S2:** Positions of the analyzed SNPs in *ABCC1*

SNP Position (Chr16) Alleles captured by analysis

rs35623 16076966 rs35621, rs35623, rs35628, rs35629, rs152029, rs152030

rs35625 16077067 rs35625

rs11866794 16077075 rs11866794

rs4148350 16077978 rs4148350

rs4148351 16078069 rs4148351, rs4148355

rs35626 16078116 rs2074086, rs35626, rs152028

rs35628 16078607 rs35628

rs4148353 16078649 rs4148353

rs4148356 16084776 rs4148356

rs11075295 16085188 rs11075295, rs7185286

rs3888565 16090546 rs3888565

rs3851711 16090588 rs4148354, rs3851711

**Supporting Information Table S3:** Sequencing primers and PCR conditions for assessment of polymorphisms in NBD1 of *ABCC1*

| **Region** | **PCR conditions** | **Primer sequences 5´ → 3´*** | **Product size** | **Mg^2+^concentration** |
| --- | --- | --- | --- | --- |
| **1** | initial hold 5´at 94°C  35 cycles: 0:30 at 94°C, 0:30 at **65**°C and 0:30 at 72°C  final hold 5´ at 72°C | F tgtaaaacgacggccagttgcacatcctgtagtcccagtt  R caggaaacagctatgaccacatgcaaacctctctccactg | 420 bp | 0.8 mM |
| **2** | initial hold 5´at 94°C  35 cycles: 0:30 at 94°C and 0:30 at **68**°C  final hold 5´ at 72°C | F tgtaaaacgacggccagttccctctctgtgaccttgaaca  R caggaaacagctatgaccacaattgaagcaggcaggattt | 897 bp | 1.6 mM |
| **3** | initial hold 5´at 94°C  35 cycles: 0:30 at 94°C, 0:30 at **63**°C and 0:30 at 72°C  final hold 5´ at 72°C | F tgtaaaacgacggccagtccctcttgccaaagcaatagtt  R caggaaacagctatgaccgcagtcatgtgaccacaaaggt | 725 bp | 1.6 mM |
| **4** | 3´ at 94°C  10 cycles: 0:30 at 94°C, 0:30 at **65 to 55**°C (touch down) and 0:30 at 72°C and 25 cycles: 0:30 at 94°C, 0:30 at **65**°C and 0:30 at 72°C  final hold 5´ at 72°C | F cgcacgtgtcctgttcttta  R catcatgttgtccaggctca | 371 bp | 1.6 mM |

The whole NBD1 and surrounding sequence was divided into four regions for SNP analysis. These regions were then amplified by PCR and sequenced.

Footnotes:

* F – forward, R – reverse primer, M13 sequence adaptors used for sequencing underlined, for sequencing of the region 4 unmodified primers were used.

**Supporting Information Table S4:** *In silico* analysis of functional significance of all studied polymorphisms in NBD1 of *ABCC1*

The functional significance of examined SNPs was analyzed *in silico* by Regulome DB (http://regulome.stanford.edu), PolyPhen-2 (http://genetics.bwh.harvard.edu/pph2) and SIFT (http://sift.jcvi.org).

| **SNP position** | **SNP** | **Result of *in silico* analysis by Regulome DB** |
| --- | --- | --- |
| chr16:16169464 | rs35623 | TF binding or DNase peak ([5](http://regulome.stanford.edu/snp/chr16/16169464)) |
| chr16:16169565 | rs35625 | TF binding + matched TF motif + DNase peak ([2c](http://regulome.stanford.edu/snp/chr16/16169565)) |
| chr16:16169573 | rs11866794 | TF binding + matched TF motif + DNase peak ([2c](http://regulome.stanford.edu/snp/chr16/16169573)) |
| chr16:16170476 | rs4148350 | TF binding or DNase peak ([5](http://regulome.stanford.edu/snp/chr16/16170476)) |
| chr16:16170567 | rs4148351 | No Data |
| chr16:16170614 | rs35626 | eQTL + TF binding / DNase peak ([1f](http://regulome.stanford.edu/snp/chr16/16170614)) |
| chr16:16171105 | rs35628 | Minimal binding evidence ([6](http://regulome.stanford.edu/snp/chr16/16171105)) |
| chr16:16171147 | rs4148353 | No Data |
| chr16:16177274 | rs4148356 | No Data |
| chr16:16177686 | rs11075295 | TF binding + DNase peak ([4](http://regulome.stanford.edu/snp/chr16/16177686)) |
| chr16:16183044 | rs3888565 | TF binding or DNase peak ([5](http://regulome.stanford.edu/snp/chr16/16183044)) |
| chr16:16183086 | rs3851711 | TF binding or DNase peak ([5](http://regulome.stanford.edu/snp/chr16/16183086)) |

Footnote: TF = transcription factor, eQTL = expression quantitative trait loci

The rs4148356 SNP was predicted to be benign with a score of 0.014 by PolyPhen-2 (http://genetics.bwh.harvard.edu/pph2) and tolerated with a score 0.30 by SIFT (http://sift.jcvi.org) programs.

**Supporting Figure S1: Flow diagram of the study**

Selection process and laboratory analyses are displayed by blue lines and statistical analyses by red lines.

**Whole study set**

**N=540 patients**

**Blood DNA**

**ABCC1 genotyping**

**N=12 SNPs**

**ABCC1 transcript levels**

**Clinical data**

**TNM, grade, receptors**

**Disease-free survival &**

**therapy**
